# Relative Intensity Noise of Gain-Switched Dual-State Lasing for an Insein(113)B Quantum Dot Laser

**DOI:** 10.3390/nano15070511

**Published:** 2025-03-28

**Authors:** Nuran Dogru, Erkan Cengiz, Hilal S. Duranoglu Tunc

**Affiliations:** 1 Electrical & Electronics Engineering, Gaziantep University, 27310 Gaziantep, Türkiye; ec11007@mail2.gantep.edu.tr; 2 Radio Frequency and Photonics Engineering, Dresden University of Technology, 01062 Dresden, Germany; hilal_sultan.duranoglu_tunc@tu-dresden.de

**Keywords:** gain switching, quantum dot, RIN, semiconductor lasers, ultrashort pulse generation

## Abstract

The RIN of an InAs/InP(113)B quantum-dot laser for direct- and cascade-relaxation models is investigated under the gain-switching condition via the application of an optical Gaussian pulse to an excited state. A new method is proposed to obtain RIN curves by eliminating the cross-correlation between noise sources. In this way, the noise sources are described independently and simulated with independent white Gaussian random variables. The results revealed that the RIN spectrum of both models was the same, apart from the fact that the cascade-relaxation model generated somewhat shorter pulses than the direct-relaxation model. Nevertheless, the direct-relaxation model had a lower RIN than that of the cascade-relaxation model. Excited- and ground-state carrier noises strongly affected the RIN spectrum, whereas the wetting-layer carrier noise had a negligible effect. In addition, the capture and escape times significantly affected the RIN spectrum. The output pulses had a long pulse width for both models due to the long pulse width of the ground-state photons. Nevertheless, applying an optical Gaussian pulse to an excited state reduced the RIN of both models and produced narrower gain-switched output pulses.

## 1. Introduction

Quantum dot (Q-Dot) lasers are a kind of semiconductor nanostructure. Q-Dot lasers have some outstanding properties, such as a low sensitivity to temperature [1], a low linewidth enhancement factor [2], optical feedback resistance, and a lower threshold current [3,4], making them attractive for various applications, particularly in the field of telecommunications [5,6,7,8]. The low-cost adjustable lasers in next-generation communication systems do not require cooling or insulation. Q-Dot lasers are considered encouraging for such areas due to their desirable characteristics. The dynamic and RIN characteristics of laser sources are crucial for communication systems, as they determine the achievable rate of a signal transporting data and the maximum distance of optical fiber links. Q-Dot lasers based on GaAs substrates generally emit radiation in the O band, and it is difficult to achieve the desired long-haul communication. In contrast, InAs Q-Dots grown on InP substrates enable the realization of laser devices operating in the C band, as the lattice mismatch in InAs/InP (~3%) is smaller than the lattice mismatch in InAs/GaAs (~7%). Therefore, we selected InAs-InP (113)B Q-Dot lasers with the InAs Q-Dot laser grown on an InP substrate, which emits at a wavelength of 1.55 μm for communication systems [4,9,10]. 

To date, many studies have been conducted on the noise of Q-Dot lasers [11,12,13]. The RIN characteristics of an InAs-InP (113)B laser were theoretically investigated under mode-locked conditions for a simple model neglecting the excited state by the authors of [14]. Low-noise pulses were experimentally obtained under a passive mode-locked InAs-InP (113)B Q-Dot laser in [15].

Gensty and Elsäßer [16] investigated the RIN properties through a semiclassical noise model for quantum cascade lasers based on a three-level rate equation. The study revealed that the RIN decreased in the low frequency limit with an increase in the pump parameter.

Sanaee and Zarifkar [17] theoretically investigated the effect of the gain compression and direct carrier switching on the RIN characteristics of 1.55 µm InAs-InP Q-Dot lasers. The results showed that the RIN level of the Q-Dot laser decreased due to the nonlinear gain, and the damping factor increased.

The modulation response and noise characteristics of a 1.55 µm InAs-InP Q-Dot laser were studied theoretically under optical pumping in [13]. The results indicated that the RIN and frequency noise decreased, while the damping factor increased.

Duan et al. [18] theoretically examined the RIN properties of the Q-Dot laser. This study demonstrated that the energy separation between the excited state (Exs) and the ground state (Grs) had a strong impact on the RIN. High energy separation is more suitable for achieving low intensity noise due to the suppression of carrier noise in the Exs. Moreover, it was shown that the carrier noise in the Exs and Grs significantly increased the RIN, while the effect of the carrier noise in the wetting layer (Wly) on the RIN was negligible.

A theoretical analysis of the RIN for InAs-InGaAs was provided in the study of Babaabasi et al. [19]. It was shown that the carrier noise in the Exs and Grs increased the RIN, and the RIN noise in the Exs and Grs increased the RIN, and the RIN decreased with the increase in the injection current.

Active/passive mode-locked, Q-switching, and gain-switching methods are well-known techniques to obtain optical short pulses. The gain-switching technique is preferred for many applications, due to its simple implementation and low manufacturing costs. Therefore, the RIN characteristics of an InAs-InP (113)B laser were investigated under the gain-switching condition in this study. To the best of our knowledge, no study has shown the intensity noise of Q-Dot lasers operating in the dual-state lasing mode under the gain-switching condition. To address this gap, we propose a novel approach to obtain low RIN for Q-Dot lasers and produce narrow optical pulses. A Gaussian pulse source is applied to the Exs of the InAs-InP Q-Dot laser, while the Wly is subjected to current injection. As mentioned before, the intensity and frequency fluctuations of Q-Dot lasers have been investigated by many researchers and, in these studies, the small-signal approximation of linearized rate equations was used. However, information is lost due to the instantaneous fluctuations in the intensity and phase; moreover, the accuracy of such an analysis under large fluctuations cannot be guaranteed. Therefore, a different method is used (instead of the small-signal approach) in order to more easily include noise sources in the rate equations and obtain accurate RIN levels. In this method, the laser rate equations are converted to new equations, as defined in [20,21], to eliminate the cross correlations between these noise sources, which enables the noise sources to be defined independently and to be simulated with independent white Gaussian random variables. This technique and the optical Gaussian pulse applied to the excited state aim to develop the performance of Q-Dot lasers and enable their application in communication systems.

## 2. Method

The Q-Dot model investigated here is based on the model explained in references [14,22,23,24,25]. The rate equations for the carriers and photons in the Grs and Exs of the Q-Dot laser were used as the basis for the direct-relaxation model (DRM) and the cascade-relaxation model (CRM). The Runge–Kutta method—a numerical integration technique commonly used for solving differential equations—was applied to numerically solve the single-mode laser rate equations. Several simplifying assumptions were made in the calculations, including neglecting the Q-Dot size fluctuations and line broadenings, the temperature effects, and the carrier loss. Applying an optical beam to the excited state allows immediate injection of carriers into the excited state, thus reducing the effect of the barrier or wetting layer. The dynamics of carriers were not taken into consideration in the barrier; direct carrier injection from the contacts to the Wly was assumed. Further, only a single discrete Grs and corresponding holes formed inside the Q-Dot were assumed, maintaining charge neutrality within each Q-Dot. Carrier and spontaneous fluctuations included in the laser rate equations were assumed as Langevin noise sources. To investigate the noise effect, facilitate control, and ensure the accuracy of the RIN analysis, a different method was used to include the noises in the differential rate equations [20,21]. To remove the cross correlations between the carrier and photon noises, new rate equations were defined, and an independent simulation using white Gaussian random variables was allowed. This approach simplifies the addition and control of noise sources. The carrier noise terms added to the Wly, Exs, and Grs equations are denoted as F_W_, F_E_, and F_G_, respectively. The photon noises in the Exs and Grs are denoted as F_SE_ and F_SG_. 

**Figure 1:**
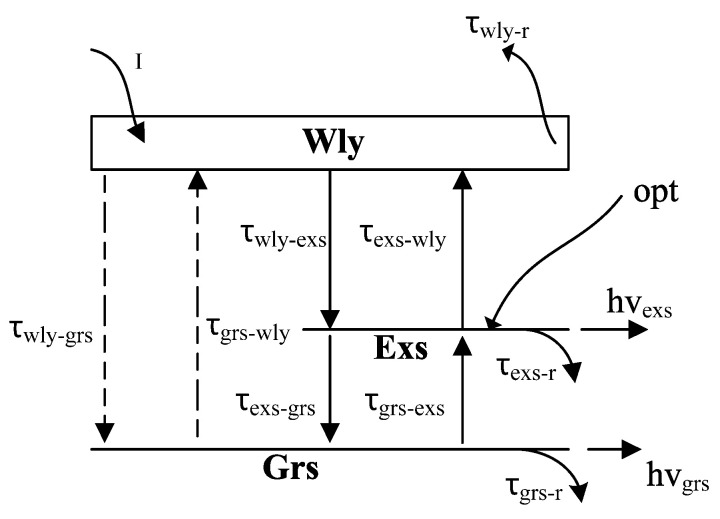
Scheme of the carrier transition process in cascade- and direct-relaxation channel models (dashed lines represent the direct model) [22].

Figure 1 shows a scheme of the carrier transition in cascade- and direct-relaxation channel models. The rate equation for the direct-relaxation channel model is determined by the following equations [22]:

(1) $$\frac{dN_{wly}}{dt}=\frac{I}{qV_{a}}-\left(1-f_{Exs}\right)\frac{N_{Wly}}{\tau_{Exs}^{Wly}}-{(1-f}_{GrS})\frac{N_{Wly}}{\tau_{Grs}^{Wly}}-\frac{N_{Wly}}{\tau_{Wly}^{spon}}+\frac{N_{Exs}}{\tau_{Wly}^{Exs}}+\frac{N_{Grs}}{\tau_{Wly}^{Grs}}+F_{W}$$

(2) $$\frac{\;\;\;\;dN_{Exs}}{dt}=\left(1-f_{Exs}\right)\frac{N_{Wly}}{\tau_{Exs}^{Wly}}+\left(1-f_{Exs}\right)\frac{N_{Grs}}{\tau_{Exs}^{Grs}}-{(1-f}_{GrS})\frac{N_{Exs}}{\tau_{Grs}^{Exs}}-\frac{N_{Exs}}{\tau_{Wly}^{Exs}}-\frac{N_{Exs}}{\tau_{Exs}^{spon}}-v_{g}\Gamma a_{Exs}\left(\frac{N_{Exs}}{2}-N_{0}\right)\frac{S_{Exs}}{\left(1+\varepsilon S_{Exs}\right)}+F_{E}+opt$$

(3) $$\frac{dN_{Grs}}{dt}={(1-f}_{GrS})\frac{N_{Wly}}{\tau_{Grs}^{Wly}}+{(1-f}_{GrS})\frac{N_{Exs}}{\tau_{Grs}^{Exs}}-\left(1-f_{Exs}\right)\frac{N_{Grs}}{\tau_{Exs}^{Grs}}-\frac{N_{Grs}}{\tau_{Grs}^{spon}}-v_{g}\Gamma a_{Grs}\left(N_{Grs}-N_{0}\right)\frac{S_{Grs}}{\left(1+\varepsilon S_{Grs}\right)}+F_{G}$$

(4) $$\frac{dS_{Exs}}{dt}=v_{g}\Gamma a_{Exs}\left(\frac{N_{Exs}}{2}-N_{0}\right)\frac{S_{Exs}}{\left(1+\varepsilon S_{Exs}\right)}+\beta \frac{N_{Exs}}{\tau_{Exs}^{spon}}-\frac{S_{Exs}}{\tau_{p}}+F_{SE}$$

(5) $$\frac{dS_{grs}}{dt}=v_{g}\Gamma a_{Grs}\left(N_{Grs}-N_{0}\right)\frac{S_{Grs}}{\left(1+\varepsilon S_{grs}\right)}+\beta \frac{N_{Grs}}{\tau_{Grs}^{spon}}-\frac{S_{Grs}}{\tau_{p}}+F_{SG}$$

The following equations demonstrate the autocorrelation and cross-correlation of the carrier and photon noises for DRM.

(6) $$V_{WW}=2D_{WW}=2\left(\frac{N_{Wly}}{\tau_{Exs}^{Wly}}\left(1-f_{Exs}\right)+\frac{N_{Wly}}{\tau_{Grs}^{Wly}}{(1-f}_{GrS})+\frac{N_{Wly}}{\tau_{Wly}^{spon}}\right)$$

(7) $$V_{EE}=2D_{EE}=2\left(\frac{N_{Exs}}{\tau_{Wly}^{Exs}}+\frac{N_{Exs}}{\tau_{Grs}^{Exs}}{(1-f}_{Grs})+\frac{N_{Exs}}{\tau_{Exs}^{spon}}+\Gamma v_{g}a_{Exs}\frac{N_{Exs}}{2}\frac{S_{Exs}}{1+\varepsilon_{Exs}S_{Exs}}\right)$$

(8) $${V_{GG}=2D}_{GG}=2\left(\frac{N_{Grs}}{\tau_{Exs}^{Grs}}{(1-f}_{Exs})+\frac{N_{Grs}}{\tau_{Wly}^{Grs}}+\frac{N_{Grs}}{\tau_{Grs}^{spon}}+\Gamma v_{g}a_{Grs}N_{Grs}\frac{S_{Grs}}{1+\varepsilon_{Grs}S_{Grs}}\right)$$

(9) $$V_{SESE}={2D}_{SESE}=2\left(\Gamma v_{g}a_{Exs}\frac{N_{Exs}}{2}\frac{S_{Exs}}{1+\varepsilon_{Exs}S_{Exs}}+\Gamma \beta \frac{N_{Exs}}{\tau_{Exs}^{spon}}\right)$$

(10) $${V_{SGSG}=2D}_{SGSG}=2\left(\Gamma v_{g}a_{Grs}N_{Grs}\frac{S_{Grs}}{1+\varepsilon_{Grs}S_{Grs}}+\Gamma \beta \frac{N_{Grs}}{\tau_{Grs}^{spon}}\right)$$

(11) $$V_{ESE}={2D}_{ESE}=-\left(\Gamma v_{g}a_{Exs}\left(\frac{N_{Exs}}{2}+N_{O}\right)\frac{S_{Exs}}{1+\varepsilon_{Exs}S_{Exs}}+\Gamma \beta \frac{N_{Exs}}{\tau_{Exs}^{spon}}\right)$$

(12) $$V_{EW}{=2D}_{EW}=-\left(\frac{N_{Exs}}{\tau_{Wly}^{Exs}}+\frac{N_{Wly}}{\tau_{Exs}^{Wly}}\left(1-f_{Exs}\right)\right)$$

(13) $${V_{EG}=2D}_{EG}=-\left(\frac{N_{Exs}}{\tau_{Grs}^{Exs}}{(1-f}_{Grs})+\frac{N_{Grs}}{\tau_{Exs}^{Grs}}{(1-f}_{Exs})\right)$$

(14) $${V_{GSG}=2D}_{GSG}=-\left(\begin{array}{c} \Gamma v_{g}a_{Grs}\left(N_{Grs}+N_{O}\right)\frac{S_{Grs}}{1+\varepsilon_{Grs}S_{Grs}}\\ +\Gamma \beta \frac{N_{Grs}}{\tau_{Grs}^{spon}} \end{array}\right)$$

(15) $$V_{GW}{=2D}_{GW}=-\left(\frac{N_{Grs}}{\tau_{Wly}^{Grs}}+\frac{N_{Wly}}{\tau_{Grs}^{Wly}}{(1-f}_{GrS})\right)$$

(16) $${V_{GE}=V_{EG}=2D}_{GE}={2D}_{UG}=-\left(\frac{N_{Exs}}{\tau_{Grs}^{Exs}}{(1-f}_{Grs})+\frac{N_{Grs}}{\tau_{Exs}^{Grs}}{(1-f}_{Exs})\right)$$

(17) $${V_{ESG}=V_{GSE}=2D}_{ESG}={2D}_{GSE}=0$$

The carrier densities of Wly, Exs, and Grs are described by $N_{Wly}$, $N_{Exs}$, and $N_{Grs}$, respectively. $f_{Exs,Grs}$ indicates the occupation probabilities of carriers in Exs and Grs. The Wly carriers are captured by the Exs or by the Grs in time, and are represented by $\tau_{Exs}^{Wly}$, $\tau_{Grs}^{Wly}$. The escape times from Exs to Wly and from Grs to Exs are defined as $\;\frac{N_{Exs}}{\tau_{Wly}^{Exs}}\;and\;\frac{N_{Grs}}{\tau_{Exs}^{Grs}},$ respectively. $\tau_{Grs}^{Exs}$ is the relaxation time for Exs to Grs. The Exs and Grs photon densities are indicated by $S_{Exs}$ and $S_{Grs}$, respectively. $a_{Exs}$ and $a_{Grs}$ used in equations are the differential gains for Exs and Grs. $v_{g}$ is the group velocity and Γ is the confinement factor. β and $N_{0}$ show the spontaneous coupling factor and the carrier density of the Q-Dot, respectively. ε represents the gain saturation parameter. Spontaneous emissions of photons from Wly, Exs, and Grs (in time) are indicated by $\tau_{Wly}^{spon}$, $\tau_{Exs}^{spon}$, and $\tau_{Grs}^{spon}$. For CRM, the terms $\frac{N_{Wly}}{\tau_{Grs}^{Wly}}$, and $\frac{N_{Grs}}{\tau_{Wly}^{Grs}}$ are neglected.

The term opt in (2) indicates the Gaussian optical pulse applied to the Exs. This term is equal to the number of photons per second per volume irradiating the Exs level in a single round trip. It is defined as $\left(1-\frac{N_{Exs}}{4N_{O}}\right)*\Gamma *v_{g}*a_{Exs}*N_{O}*\frac{P\left(\frac{2L}{v_{g}}\right)}{E_{Exs}*V_{a}*q}.$ *P* is the peak power of the applied optical beam.

To generate simultaneously the cross-correlated noises $F_{W},\;F_{E},F_{G},\;F_{SE}$, and $F_{SG}$, the laser rate equations in [13] are transformed into five different equations of the photon densities: $S_{E}$, $S_{G}$, Wly carrier density, $N_{W}$ and variables determined as $k_{1}S_{E}+m_{1}N_{W}+n_{1}N_{G}+N_{E}$ for the excited state carrier density and $k_{2}S_{G}+m_{2}N_{W}+n_{2}N_{E}+N_{G}$ for the ground state carrier density.

(18) $$\frac{\;dN_{Wly}}{dt}=\frac{I}{qV}+\frac{N_{Exs}}{\tau_{Wly}^{Exs}}+\frac{N_{Grs}}{\tau_{Wly}^{Grs}}-\frac{N_{Wly}}{\tau_{Exs}^{Wly}}\left(1-f_{Exs}\right)+\frac{N_{Wly}}{\tau_{Grs}^{Wly}}{(1-f}_{GrS})-\frac{N_{Wly}}{\tau_{Wly}^{spon}}+F_{W}$$

(19) $$\frac{dS_{Exs}}{dt}=\Gamma v_{g}a_{Exs}\left(\frac{N_{Exs}}{2}-N_{O}\right)\frac{S_{Exs}}{1+\varepsilon_{Exs}S_{Exs}}-\frac{S_{Exs}}{\tau_{p}}+\beta \frac{N_{Exs}}{\tau_{Exs}^{spon}}+F_{SE}$$

(20) $$\frac{dS_{Grs}}{dt}=\Gamma v_{g}a_{Grs}\left(N_{Grs}-N_{O}\right)\frac{S_{Grs}}{1+\varepsilon_{Grs}S_{Grs}}-\frac{S_{Grs}}{\tau_{p}}+\beta \frac{N_{Grs}}{\tau_{Grs}^{spon}}+F_{SG}$$

(21) $$\frac{d\left\{{k_{1}S_{E}+m_{1}N_{W}+n_{1}N_{G}+N}_{E}\right\}}{dt}=k_{1}\left\{\frac{dS_{Exs}}{dt}\right\}+m_{1}\left\{\frac{\;dN_{Wly}}{dt}\right\}+n_{1}\left\{\frac{d\left\{{k_{2}S_{G}+m_{2}N_{W}+n_{2}N_{E}+N}_{G}\right\}}{dt}\right\}+\;\left\{\frac{dN_{Exs}}{dt}\right\}+\left\{k_{1}F_{SE}\left(t\right)+m_{1}F_{W}\left(t\right)+n_{1}F_{G}\left(t\right)+F_{E}(t)\right\}$$

(22) $$\frac{d\left\{{k_{2}S_{G}+m_{2}N_{W}+n_{2}N_{E}+N}_{G}\right\}}{dt}=k_{2}\left\{\frac{dS_{Grs}}{dt}\right\}+m_{2}\left\{\frac{\;dN_{Wly}}{dt}\right\}+n_{2}\left\{\frac{d\left\{{k_{1}S_{E}+m_{1}N_{W}+n_{1}N_{G}+N}_{E}\right\}}{dt}\right\}+\;\left\{\frac{dN_{Grs\_}}{dt}\right\}+\left\{k_{2}F_{SG}\left(t\right)+m_{2}F_{W}\left(t\right)+n_{2}F_{E}\left(t\right)+F_{G}(t)\right\}$$

Here, $k_{1,},\;{k_{2},\;m}_{1},\;m_{2},n_{1}{,n}_{2}\;$ are the real numbers defined as follows:

(23) $$k_{1}=-\frac{V_{ESE}}{V_{SESE}},\;m_{1}=-\frac{V_{EW}}{V_{WW}},\;n_{1}=-\frac{V_{EG}}{V_{GG}}$$

(24) $$k_{2}=-\frac{V_{GSG}}{V_{SGSG}},\;m_{2}=-\frac{V_{GW}}{V_{WW}},\;n_{2}=-\frac{V_{GE}}{V_{EE}}$$

There are no cross-correlations among the noise functions $F_{W}(t),F_{SE}(t),F_{SG}(t)$, $\left[k_{1}F_{SE}\left(t\right)+m_{1}F_{W}\left(t\right)+n_{1}F_{G}\left(t\right)+F_{E}\left(t\right)\right]$ and ${[k}_{2}F_{SG}\left(t\right)+m_{2}F_{W}\left(t\right)+n_{2}F_{E}\left(t\right)+F_{G}(t)]$ and they are mutually orthogonal. Therefore, they can be handled independently. The auto-correlations between the new random functions can be expressed as follows:

(25) $$\langle \left\{k_{1}F_{SE}\left(t\right)+m_{1}F_{W}\left(t\right)+n_{1}F_{G}\left(t\right)+F_{E}\left(t\right)\right\}.\left\{k_{1}F_{SE}\left(t^{\prime }\right)+m_{1}F_{W}\left(t^{\prime }\right)+n_{1}F_{G}\left(t^{\prime }\right)+F_{E}\left(t^{\prime }\right)\right\}\rangle \;=\left\{k_{1}V_{SESE}+m_{1}V_{EW}+n_{1}V_{EG}+{2m}_{1}n_{1}V_{GW}+V_{EE}\right\}\delta \left(t-t^{\prime }\right)\;\;\;\;\;\;\;\;\;\;$$

(26) $$\langle \left\{k_{2}F_{SG}\left(t\right)+m_{2}F_{W}\left(t\right)+n_{2}F_{E}\left(t\right)+F_{G}(t)\right\}\cdot \left\{k_{2}F_{SG}\left(t^{\prime }\right)+m_{2}F_{W}\left(t^{\prime }\right)+2F_{E}\left(t^{\prime }\right)+F_{G}(t^{\prime })\right\}\rangle \;=\left\{k_{2}V_{SGSG}+m_{2}V_{GW}+n_{2}V_{GE}+{2m}_{2}n_{2}V_{EW}+V_{GG}\right\}\delta \left(t-t^{\prime }\right)\;\;\;\;\;\;\;$$

The carrier noises $F_{E}$ and $F_{G}\;$ for Exs and Grs can be found now from the following:

(27) $$F_{E}\left(t\right)=\left\{k_{1}F_{SE}\left(t\right)+m_{1}F_{W}\left(t\right)+n_{1}F_{G}\left(t\right)+F_{E}(t)\right\}-k_{1}F_{SE}\left(t\right)-m_{1}F_{W}\left(t\right)-n_{1}F_{G}\left(t\right)$$

(28) $$F_{G}\left(t\right)=\left\{k_{2}F_{SG}\left(t\right)+m_{2}F_{W}\left(t\right)+n_{2}F_{E}\left(t\right)+F_{G}(t)\right\}-k_{2}F_{SG}\left(t\right)-m_{2}F_{W}\left(t\right)-n_{2}F_{E}\left(t\right)$$

The delta functions are numerically solved as follows:

(29) Fi(t)Fj(t′)=Vij∆t   for   t−t′<∆t                    0       for      t−t′>∆t

∆t indicates the sampling time. Noise sources are expressed as follows:

(30) $$F_{W}=\sqrt{\frac{V_{WW}}{∆t\times V}}g_{W}$$

(31) $$\left\{k_{1}F_{SE}\left(t\right)+m_{1}F_{W}\left(t\right)+n_{1}F_{G}\left(t\right)+F_{E}(t)\right\}=\sqrt{\frac{k_{1}V_{SESE}+m_{1}V_{EW}+n_{1}V_{EG}+{2m}_{1}n_{1}V_{GW}+V_{EE}}{∆t\times V}}g_{E}$$

(32) $$\left\{k_{2}F_{SG}\left(t\right)+m_{2}F_{W}\left(t\right)+n_{2}F_{E}\left(t\right)+F_{G}(t)\right\}=\sqrt{\frac{k_{2}V_{SGSG}+m_{2}V_{GW}+n_{2}V_{GE}+{2m}_{2}n_{2}V_{EW}+V_{GG}}{∆t\times V}}g_{G}$$

(33) $$F_{SE}=\sqrt{\frac{V_{SESE}}{∆t\times V}}g_{SE}$$

(34) $$F_{SG}=\sqrt{\frac{V_{SGSG}}{∆t\times V}}g_{SG}$$

Thus, the laser rate equations in [13] can be integrated using the $F_{W},\;F_{E},F_{G},\;F_{SE},\;$ and $F_{SG}$, which means that Equations (13)–(17) are integrated by (25)–(29).$\;g_{W}$, $g_{E}$, $g_{G}$,$\;g_{SE},\;$ and $g_{SG}$ are supposed to be independent random numbers. They form the Gaussian probability distribution functions with zero mean values of $\langle g_{W}\rangle =$ $\langle g_{E}\rangle =\langle g_{G}\rangle =$ $\langle g_{SE}\rangle =\langle g_{SG}\rangle =$0 and unit variances $\langle g_{W}^{2}\rangle =$ $\langle g_{E}^{2}\rangle =\langle g_{G}^{2}\rangle =$ $g_{SE}^{2}=\langle g_{SG}^{2}\rangle =1$.

RIN is calculated as follows:

(35) $$RIN(f)=\frac{{\left|{∆S}_{Exs,\;Grs}\right|}^{2}}{S_{Exs,\;Grs}^{2}}$$

ΔS is the change in the photon density of Exs and Grs in the frequency analysis. The average of photon density is represented by S.

## 3. Results

In this section, the RIN spectrum of 1.55 μm InAs-InP (113)B Q-Dot laser was investigated under the gain-switching condition for DRM and CRM. The parameters given in Table 1 are from [14,22,23]. The gain saturation parameter was taken as 1 × 10^–16^ cm^3^ for both Grs and Exs. Since the gain-switched pulses were generated at frequencies between 400 MHz and 1 GHz, the RIN spectrum was also examined in this frequency range. AC current I was described as follows:

$$I(t)=I_{rf}\;2\;(|cos(2\Pi ft)|-cos(2\Pi ft))$$

where f is the frequency and Irf is the amplitude of the applied current.

Calculated threshold currents for DRM and CRM are given in Table 2.

**Figure 2:**
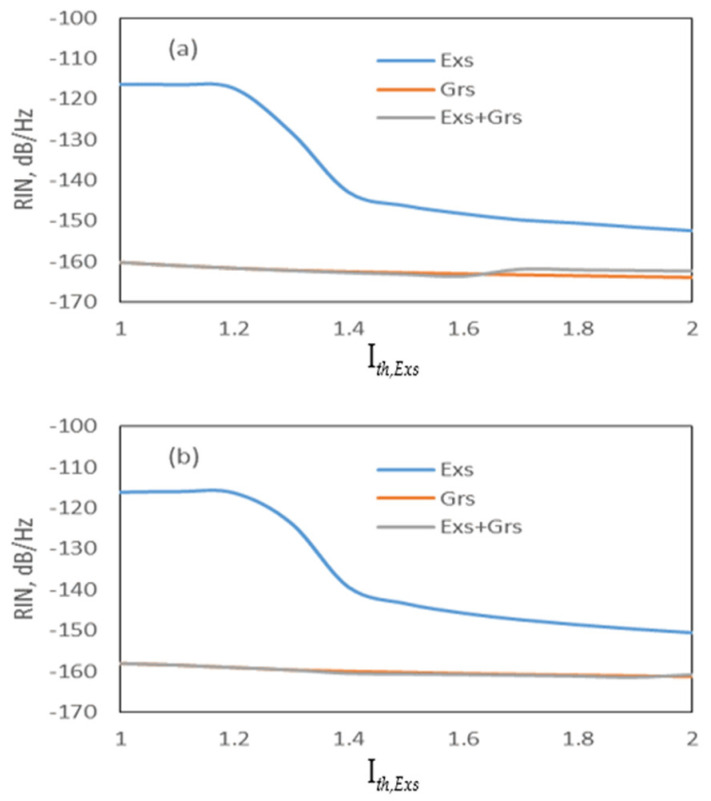
RIN spectrum as a function of I*th_,Exs_* (**a**) DRM and (**b**) CRM.

**Figure 3:**
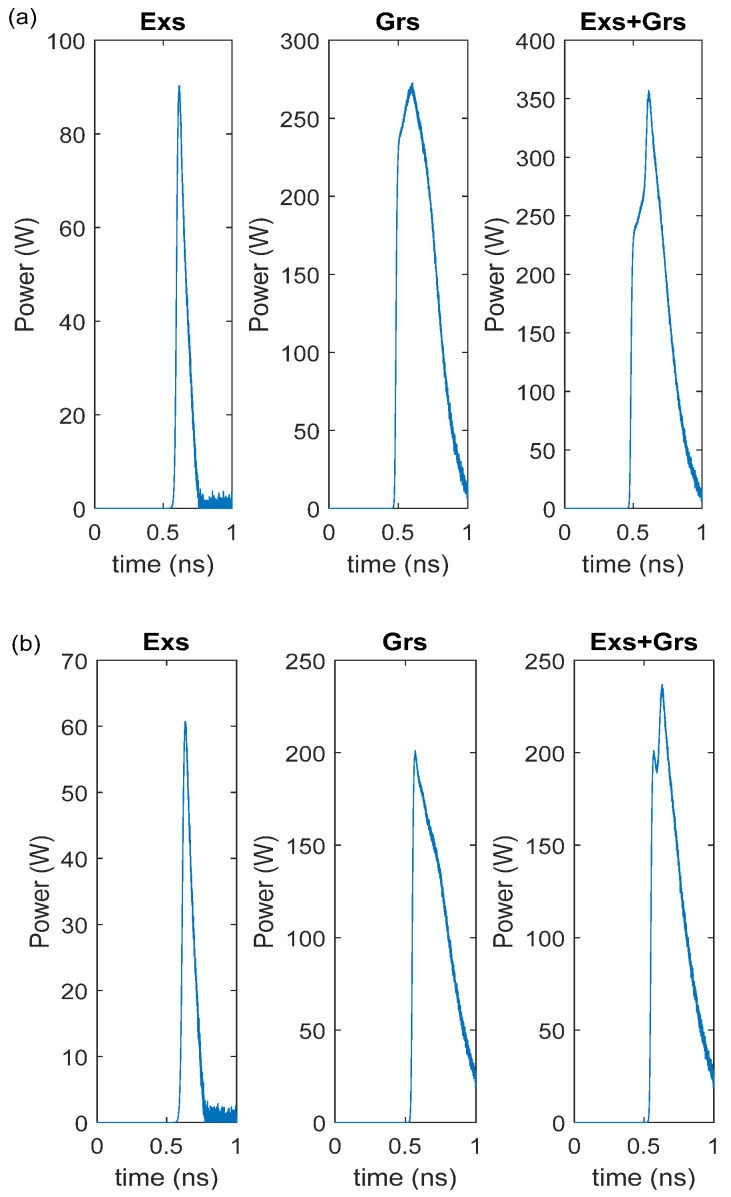
Output pulses at 1.6 × I_th,Exs_ with noise (**a**) DRM, (**b**) CRM.

Figure 2 shows the RIN of the curves of the Exs, Grs, and Exs + Grs as a function of the applied current. The Grs + Exs represents the simultaneous emission from both Grs and Exs. It is seen that the curves related to the DRM and CRM spectra almost coincide. When the injected current amplitude is higher than the threshold current of the Grs, the RIN drops with an increase in the current for the Exs, and it remains approximately constant for the Grs and Exs + Grs. Therefore, the gain-switched pulses are produced at all currents for the Grs, whereas the pulses are generated at only low values of RIN for the Exs. The decrease in RIN with the increase in the current was also observed in [26,27]. As seen in Figure 2, since the peak power value of the DRM is greater than the CRM, as the current increases, the DRM has an approximately 2 dB lower RIN than the CRM. Because of the dominant Grs emission over the Exs emission, as seen in Figure 3, the pulse width of the generated pulses is wide (see Table 3) due to the Grs photons having long pulse widths [22,28,29].

**Figure 4:**
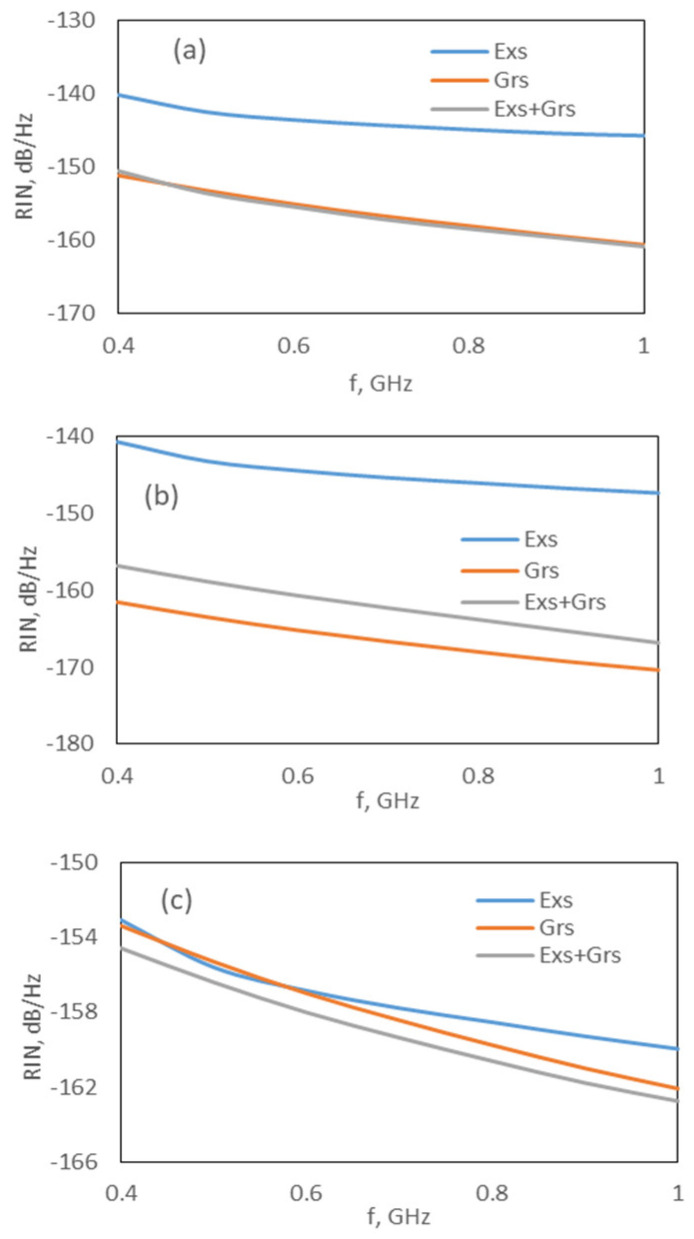
RIN spectrum at 1.6 × I_th,Exs_ for the DRM: (**a**) all noises are included, (**b**) without F_G_ and (**c**) without F_E_.

**Figure 5:**
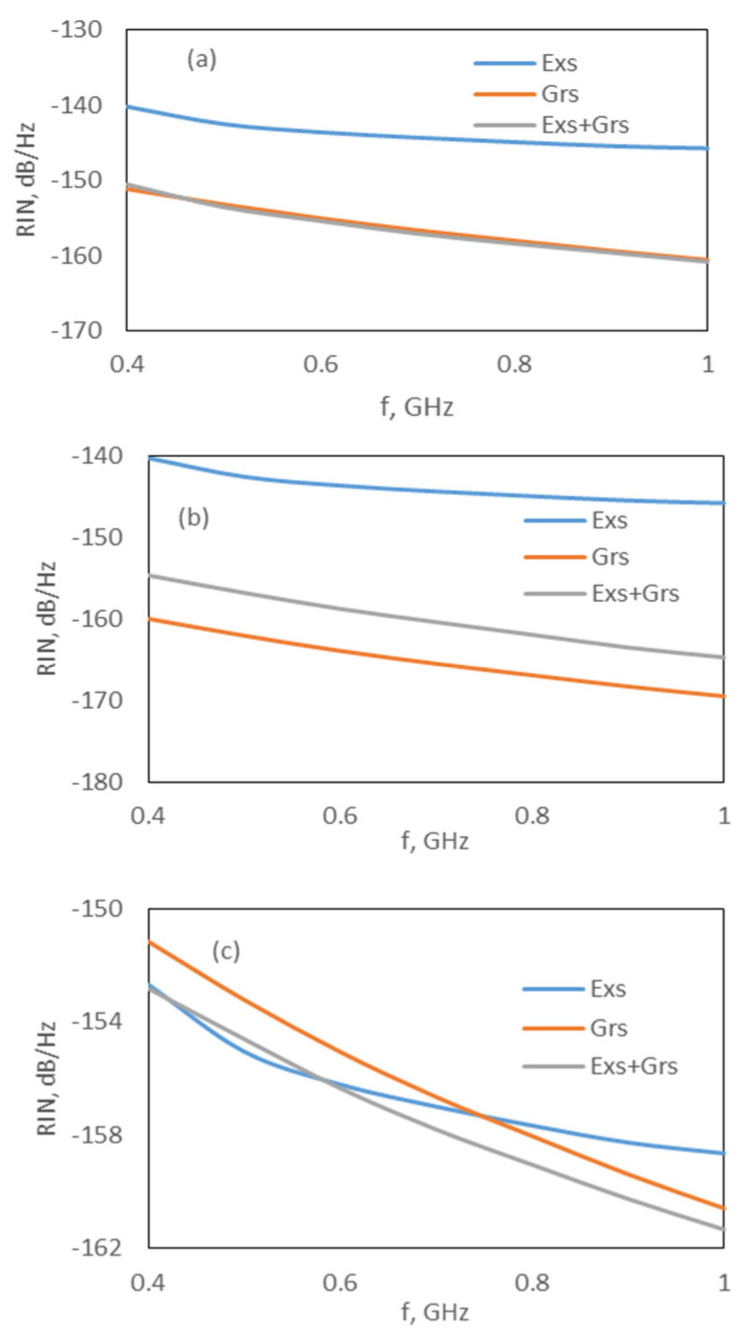
RIN spectrum at 1.6 × I_th,Exs_ for CRM; (**a**) all noise is included, (**b**) without F_G_ and (**c**) without F_E_.

Although the effect of the carrier noise in the wetting layer, F_W_, is negligible on the RIN spectrum, our simulation results also indicated that the Exs and Grs carrier noises represented by F_E_ and F_G_ strongly affect the RIN spectrum. The effects of F_E_ and F_G_ can be observed in Figure 4 and Figure 5. For both models, the RIN of the Exs is not affected in the absence of F_G_, whereas the RIN of the Grs and Exs + Grs decreases by approximately 9 dB and 4 dB, respectively (Figure 4a,b and Figure 5a,b). However, in the absence of F_E_, the RIN of the Grs is not affected, while the RIN of the Exs decreases nearly by 12 dB, and the RIN of the Exs + Grs decreases around 1 or 2 dB at low frequencies and remains approximately constant with an increase in the frequency (see Figure 4a–c and Figure 5a–c). These results showed that the carrier noise sources F_E_ and F_G_ should be taken into account whenever the dynamics of a Q-Dot laser are investigated. A similar behavior was also reported in [26]. In [26,27], it was shown that the RIN spectra of the single-mode InAs-InP(113)B Q-Dot laser were approximately constant up to 1 GHz. In addition, similar results were obtained for the multi-modal Q-Dot lasers [11,30].

**Figure 6:**
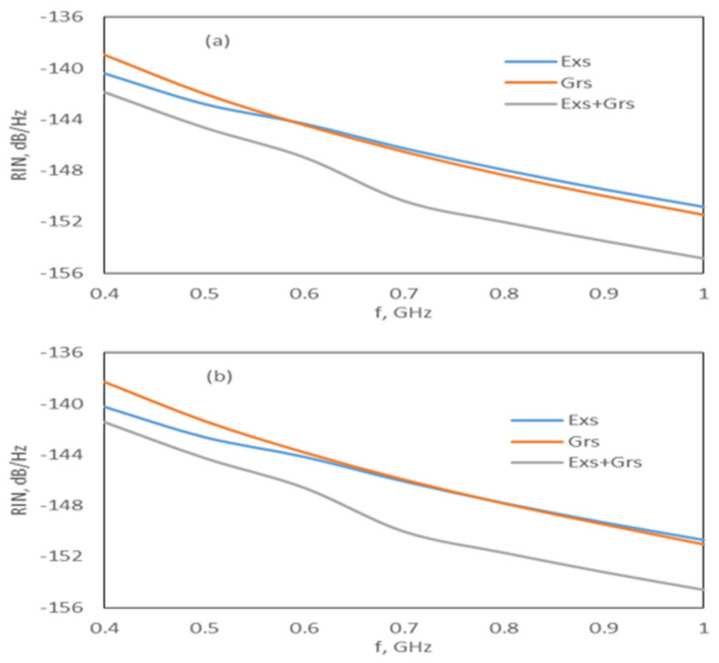
RIN spectrum under the Gaussian pulse beam for I_rf_ = 5 mA: (**a**) DRM and (**b**) CRM.

**Figure 7:**
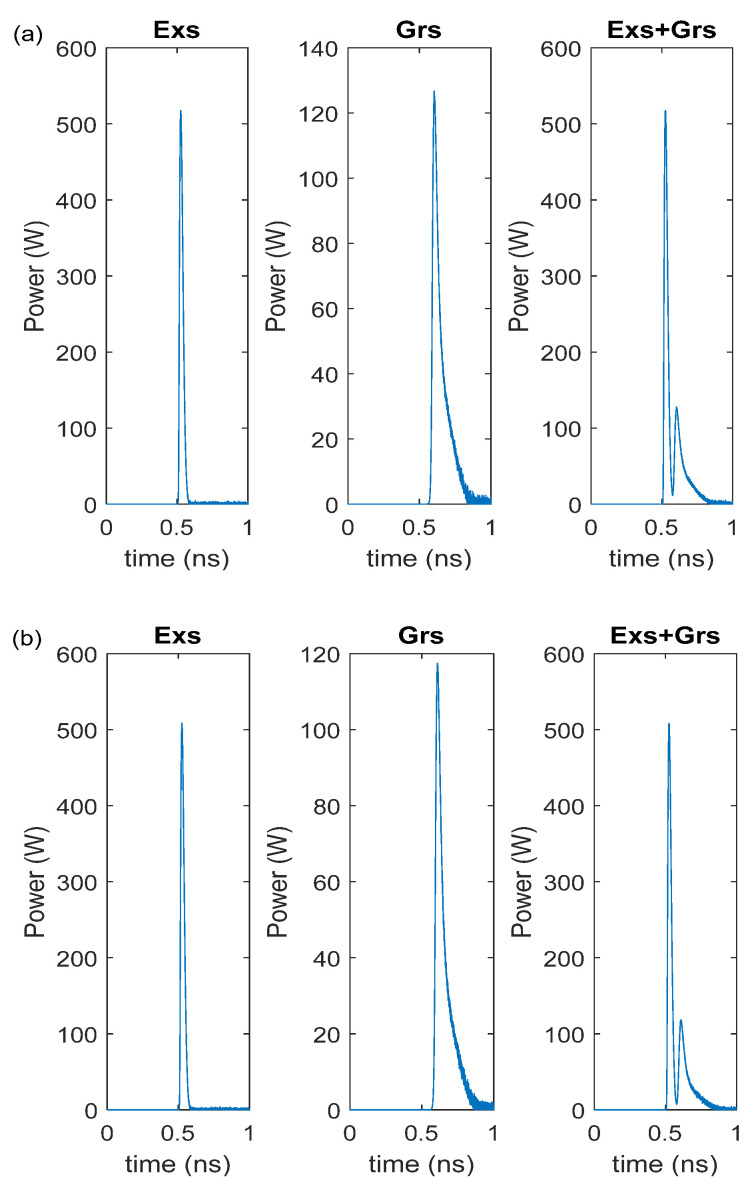
Output pulses under the Gaussian pulse beam for I_rf_ = 5 mA: (**a**) DRM and (**b**) CRM.

Given that the width of the Grs photons is long [13,22,23,24], as stated before, the output pulse of the (Exs + Grs) is wider. In addition, while the Grs photon density gradually decays after obtaining the maximum value, the Exs output power decays fast after obtaining the maximum value. Therefore, the long pulses in the InAs-InP (113)B lasers are due to the InP ground-state emission. Therefore, to reduce the RIN level and to produce shorter pulses with a high power, a Gaussian pulse was applied to the Exs. Figure 6 shows the RIN level under the Gaussian pulse with a peak power of 10 mW and I_rf_ current of 5 mA. The RIN level was low for the Exs, Grs, and Exs + Grs, even when the peak current was very low. Gain-switched output pulses were generated for both the Exs and Grs radiations. Nevertheless, the Exs radiation was dominant over the Grs; therefore, the output pulse width was narrow, owing to the Exs radiation (see Table 4 and Figure 7).

**Figure 8:**
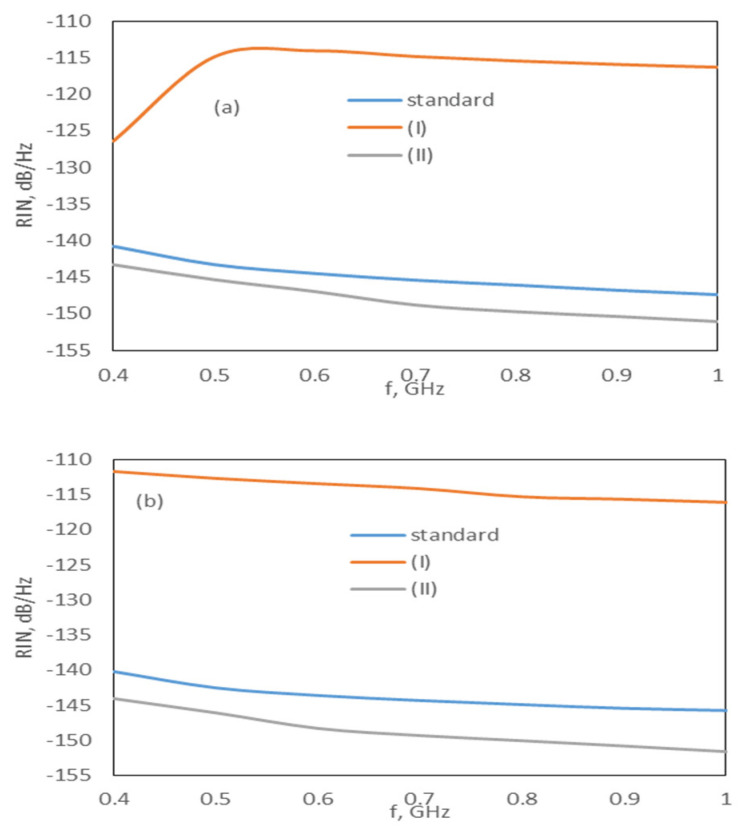
Effect of case I and II on the RIN spectrum of Exs at 1.6 × I_th,Exs_ for the (**a**) DRM and (**b**) CRM.

Furthermore, our results showed that the relaxation and capture times significantly affected the RIN spectrum and output pulses, while β, $\tau_{Wly}^{spon},\tau_{Wly}^{spon},\tau_{Grs}^{spon}$ have a slight effect on the spectrum and pulses. To demonstrate the effect of capture and relaxation times, we investigated the two cases described below.

(I) Slow capture and fast relaxation ($\tau_{Exs}^{Wly}>\tau_{Grs}^{Exs}$): To ensure this, the phonon and Auger terms A_wly_ and C_wly_ were taken as 0.7 × 10^10^ s^−1^ and 2 × 10^−9^ cm^−3^s^−1^, respectively. For both models, in this case, the Exs and Grs threshold currents increased; however, as the increase in the Exs was higher, while the increase in the Grs was very small, there was no change in the RIN spectra of the Grs and Exs + Gr, while the RIN of the Exs value increased, as observed in Figure 8a,b. Gain-switched pulses from the Exs radiation could not be generated at any frequency.
(II) Fast capture and slow relaxation ($\tau_{Exs}^{Wly}<\tau_{Grs}^{Exs}$): To ensure this, the phonon and Auger terms A_exs_ and C_exs_ were taken as 0.7 × 10^10^ s^−1^ and 6 × 10^−8^ cm^−3^s^−1^, respectively. In this case, while the Grs threshold current did not change for both models, the Exs threshold current decreased. As the Exs threshold current decreased, the RIN of the Exs decreased by 3 dB for the DRM and 4 dB for the CRM (see Figure 8). There was no significant change in the RIN of the Grs and Exs + Grs for the DRM, while the RIN of the Grs and Exs + Grs increased by 2 dB for the CRM. Gain-switched pulses originating from the Exs and Grs radiation were obtained at all frequencies.

As a result, the DRM and CRM of the InAs-InP(113)B Q-Dot lasers exhibited the same RIN behavior, except that the CRM generated slightly shorter pulses than the DRM. However, the DRM had a lower RIN than the CRM. The results also confirmed that the RIN curves obtained with the method we used here were similar to the results obtained with the small-signal approach. The RIN values we obtained verified the values measured and calculated in the experiments so far [11,26,27,31,32,33]. Moreover, the RIN for both GaAs and InP-based Q-Dot lasers was measured to be approximately −160 dB/Hz, whereas the RIN level for Q-Dot lasers grown on Ge and Si substrates was found to be approximately −120 and −150 dB/Hz [12,34,35,36,37]. Our calculated RIN results of approximately −150 dB/Hz confirmed the numerical and experimental results [17,38].

## 4. Conclusions

In this work, to the best of our knowledge, the relative intensity noise of a gain-switched 1.55 μm InAs-InP laser was theoretically examined using a different method for the first time, and short pulses from direct-relaxation and cascade-relaxation models were obtained by applying a Gaussian optical pulse to the excited state of the laser. The purpose of this method was to eliminate the cross correlations between the noise sources to describe them independently and simulate them with independent white Gaussian random variables. The results showed that the RIN results obtained using our proposed method are similar to previously published numerical and experimental results.

The RIN spectrum of both models was the same, while the cascade-relaxation model generated slightly shorter pulses than those of the direct-relaxation model. Nevertheless, the direct-relaxation model had a lower RIN than that of the cascade-relaxation model. It was also found that while the ground- and excited-state carrier noise had a significant effect on the RIN spectrum, the wetting-layer carrier noise had a negligible effect.

Our results showed that after applying an optical Gaussian pulse to the excited state of an InAs-InP (113)B quantum dot laser, a small RIN level was obtained for both the direct- and cascade-relaxation models, even when the applied peak current was very low. In addition, under the Gaussian pulse beam, the gain-switched output pulses had a high output power and narrower pulse width.

Furthermore, the obtained results showed that in the case of slow capture and fast relaxation, the RIN of the Exs increased significantly, while in the case of fast capture and slow relaxation, the RIN of the excited state decreased slightly. In both cases, there was no significant change in the RIN spectra of the ground and (ground + excited).

In summary, the low RIN levels for direct- and cascade-relaxation models under Gaussian optical pulses make Q-Dot lasers prospective resources for use in various areas such as a wide range of high-speed optical communications, optical interconnection, and signal processing applications, as well as biomedical imaging.

## Figures and Tables

**Table 1:** Q-Dot laser parameters [22].

Cavity length	L = 0.245 cm
Cavity width	w = 12 µm
Differential gain for Grs	a_grs_ = 4.6 × 10^–14^ cm^2^
Differential gain for Exs	a_exs_ = 9.2 × 10^–14^ cm^2^
Confinement factor	Γ = 0.025
Quantum dot density	N_o_ = 6 × 10^16^ cm^−3^
Refractive index	n_r_ = 3.27
Cavity internal loss	α_int_ = 6 cm^−1^
Mirror reflectivity	R_1_ = 0.95, R_2_ = 0.05
Gain compression factor	ε_grs,exs_ = 1 × 10^–16^ cm^3^
Spontaneous emission, Wly	$\tau_{Wly}^{spon}$= 500 ps
Spontaneous emission, Exs	$\tau_{Exs}^{spon}$= 500 ps
Spontaneous emission, Grs	$\tau_{Grs}^{spon}$= 1.2 ns
Photon lifetime	τ_p_ = 8.92 ps
Spontaneous coupling factor	β = 1 × 10^–4^
Wly emission energy	E_wly_ = 1.05 eV
Exs emission energy	E_exs_ = 0.840 eV
Grs emission energy	E_grs_ = 0.792 eV
Wly phonon relaxation	A_wly_ = 1.35 × 10^10^ s^−1^
Wly Auger coefficient	C_wly_ = 5 × 10^−9^ cm^3^s^−1^
Wly phonon relaxation	A_exs_ = 1.5 × 10^10^ s^−1^
Exs Auger coefficient	C_exs_ = 9 × 10^−8^ cm^3^s^−1^
Degeneracy, Grs, Exs, Wly	µ_grs,exs,wly_ = 2, 4, 10
Operating frequency	f = 1 GHz
Wavelength	λ = 1.55 µm

**Table 2:** Threshold currents of Exs, Grs and Exs + Grs for DRM and CRM.

	I*th_,Exs_*	I*th*_,*Grs*_	I*th_,Exs+Grs_*
DRM	61 mA	8 mA	8 mA
CRM	51 mA	9 mA	9 mA

**Table 3:** Pulse width and peak Power of output pulses at 1.6 × _I_th for (**a**) DRM and (**b**) CRM.

**(a)**	**Exs**	**Grs**	**Exs + Grs**
Without noise	77 ps 89 mW	303 ps 269 mW	265 ps 354 mW
With noise	74 ps 90 mW	298 ps 273 mW	262 ps 357 mW
**(b)**	**Exs**	**Grs**	**Exs + Grs**
Without noise	80 ps 60 mW	259 ps 200 mW	229 ps 234 mW
With noise	74 ps 61 mW	252 ps 201 mW	226 ps 236 mW

**Table 4:** Pulse width and peak power of output pulses under the Gaussian pulse beam for I_RF_ = 5 mA (**a**) DRM, (**b**) CRM.

**(a)**	**Exs**	**Grs**	**Exs + Grs**
Without noise	29 ps 517 mW	50 ps 126 mW	29 ps 517 mW
With noise	29 ps 518 mW	51 ps 126 mW	29 ps 518 mW
**(b)**	**Exs**	**Grs**	**Exs + Grs**
Without noise	29 ps 508 mW	50 ps 117 mW	29 ps 508 mW
With noise	29 ps 510 mW	50 ps 117 mW	29 ps 510 mW

## Data Availability

The original contributions presented in this study are included in the article. Further inquiries can be directed to the corresponding author.
